# A Functional SNP in the Promoter of *LBX1* Is Associated With the Development of Adolescent Idiopathic Scoliosis Through Involvement in the Myogenesis of Paraspinal Muscles

**DOI:** 10.3389/fcell.2021.777890

**Published:** 2021-11-30

**Authors:** Leilei Xu, Zhenhua Feng, Zhicheng Dai, Wayne Y. W. Lee, Zhichong Wu, Zhen Liu, Xu Sun, Nelson Tang, Jack Chun-Yiu Cheng, Yong Qiu, Zezhang Zhu

**Affiliations:** ^1^ Division of Spine Surgery, Department of Orthopedic Surgery, Nanjing Drum Tower Hospital, The Affiliated Hospital of Nanjing University Medical School, Nanjing, China; ^2^ Joint Scoliosis Research Center of The Chinese University of Hong Kong and Nanjing University, Nanjing/Hong Kong, China; ^3^ SH Ho Scoliosis Research Laboratory, Department of Orthopaedics and Traumatology, The Chinese University of Hong Kong, Hong Kong, China; ^4^ Li Ka Shing Institute of Health Sciences, The Chinese University of Hong Kong, Hong Kong, China; ^5^ Department of Chemical Pathology, The Chinese University of Hong Kong, Hong Kong, China

**Keywords:** gene variant, LBX1 gene, adolescent idiopathic scoliosis (AIS), myogenesis, paraspinal muscle

## Abstract

Previous studies have shown that *LBX1* is associated with adolescent idiopathic scoliosis (AIS) in multiple populations. For the first time, rs1322330 located in the putative promoter region of *LBX1* was found significantly associated with AIS in the Chinese population [*p* = 6.08 × 10^–14^, odds ratio (OR) = 1.42, 95% confidence interval of 1.03–1.55]. Moreover, the luciferase assay and electrophoretic mobility shift assay supported that the allele A of rs1322330 could down-regulate the expression of *LBX1* in the paraspinal muscles of AIS. In addition, silencing *LBX1* in the myosatellite cells resulted in significantly inhibited cell viability and myotube formation, which supported an essential role of *LBX1* in muscle development of AIS. To summarize, rs1322330 may be a novel functional SNP regulating the expression of *LBX1*, which was involved in the etiology of AIS possibly *via* regulation of myogenesis in the paraspinal muscles.

## Introduction

As a multifactorial disease, the etiology of adolescent idiopathic scoliosis (AIS) remains poorly understood (Murray and Bulstrode, 1996; Kouwenhoven and Castelein, 2008). Family aggregation of AIS has been well documented in earlier literatures (Miller et al., 2005; Miller, 2007). Thus, it was speculated that AIS could be a complex polygenic disease influenced by different loci. Following this speculation, genetic factors have been extensively investigated in the past decades. Initially, genome-wide linkage analysis and candidate gene association studies were applied to unveil the genetic background of AIS (Miller et al., 1996; Inoue et al., 2002; Morcuende et al., 2003; Liu et al., 2010). However, both methods had low efficiency to provide accurate and replicable results (Takahashi et al., 2011b).

In recent years genome-wide association studies (GWASs) were used to investigate the genetic factors involved in the development of AIS (Sharma et al., 2011; Takahashi et al., 2011a; Kou et al., 2013; Ogura et al., 2015; Sharma et al., 2015; Zhu et al., 2015; Zhu et al., 2017). Takahashi et al. (2011b) performed the first GWAS in the Japanese population and reported a remarkable association between rs11190870 of *LBX1* and AIS. Subsequently, replication studies validated that rs11190870 of *LBX1* are associated with AIS in different populations (Jiang et al., 2013; Grauers et al., 2015; Nada et al., 2018). *LBX1* has been shown to play a key role in the migration of muscle precursors in mice (Brohmann et al., 2000; Gross et al., 2000). Guo et al. (2016) reported a novel pathological feature of *LBX1* that its overexpression could cause lateral body curvature *via* the impairment of *Wnt* signaling. Interestingly, AIS patients were reported to have asymmetric expression of *LBX1* between the bilateral paraspinal muscles (Xu et al., 2020). Besides, remarkably asymmetric proportion of type I/II fibers between the bilateral sides of paraspinal muscles was also reported in AIS patients (Ford et al., 1984; Wajchenberg et al., 2015; Shao et al., 2020). However, these observations may represent secondary changes after onset of scoliosis, instead of the primary etiology. To date, there is still a paucity of knowledge concerning the functional role of *LBX1* in the myogenesis of AIS patients. Moreover, the role of genetic variants in the regulatory mechanism of *LBX1* remains obscure.

As a non-coding single-nucleotide polymorphism (SNP), rs11190870 was located about 10 kb downstream of *LBX1*. Through fine-mapping of a 40-kb region around rs11190870, we aimed to pinpoint the functional variant that could physically regulate the promoter region of *LBX1* and uncover the underlying regulatory mechanism by *in vivo* experiments. Furthermore, we aimed to investigate the role of *LBX1* in the myogenesis of AIS patients.

## Materials and Methods

### Subjects

Under the approval of the Ethics Committee of the Nanjing University Medical School-Affiliated Nanjing Drum Tower Hospital, female AIS patients who visited our Joint Scoliosis Center between July 2010 and September 2017 were reviewed for the eligibility to be included in this study. The healthy participants were recruited during the physical examinations for college admission. All the control subjects were excluded to have scoliosis through the Adam’s forward bend test by a senior spine surgeon (YQ). The current case–control association study was composed of 1,980 patients and 2,499 controls from Chinese Han population. Stage 1 was comprised of 980 patients and 1,499 controls that had been recruited in our previous GWAS. The replication stage was comprised of 1,000 patients and 1,000 controls. Informed consent was obtained from the guardians of the participants. Baseline characteristics including initial age and curve magnitude were collected from the medical record.

### Imputation Analysis

Genotype imputation was performed with MaCH-Admix software (Liu et al., 2013). The linkage disequilibrium (LD) and haplotype information of the 1000 Genomes Project (phased CHB and CHS data; March 2012 release) were used as the reference, which covered about 40 kb around rs11190870 (Elnitski et al., 2007). After imputation, SNPs with a low imputation quality (*R* ^2^ < 0.30) or with minor allele frequency (MAF) <0.10 were excluded from the output files. PLINK v1.90 was used to calculate the association of the SNPs with AIS with logistic regression model (Purcell et al., 2007). The online tool LocusZoom was used to plot the genomic regions (Pruim et al., 2010). According to the results of the imputation analysis, the most significant SNP rs1322330 in the promoter region of *LBX1* was selected for further replication in 1,000 cases and 1,000 controls.

### Genotyping

SNP rs1322330 was genotyped using TaqMan SNP Genotyping Assay. Interpretation of genotyping assay was performed by ABI 7900HT Sequence Detection System (Applied Biosystems, Foster City, CA). Thirty percent of the samples were randomly selected to validate the reliability of the genotyping results.

### Sample Collection

Blood sample was collected for all participants. Genomic DNA was subsequently extracted using a commercial kit (QIAGEN, Tokyo, Japan) according to the standard protocol. Paraspinal muscles were collected from 134 female AIS patients with main thoracic curve during the surgical intervention. Besides, 28 congenital scoliosis (CS) patients with AIS-like thoracic curve who underwent correction surgery in our clinics were included as the control group. Deep paraspinal muscle biopsies of 1.5 × 1.5 × 1.5 cm^3^ were collected at the bilateral side of the proximal vertebra of the curve for all the patients. The informed consent of sample collection was obtained from the guardians of all patients.

### Luciferase Assay

HEK293T cells were grown in 48-well cell culture plates for 24 h. They were then transiently transfected with a promoterless luciferase vector (pGL4.19-basic) (Promega, United States) or with a pGL4.19-basic vector with the *LBX1* promoter fragment (−2,060 to −120) harboring the rs1322330 A-allele or G-allele (construct rs1322330_A-allele or construct rs1322330_G-allele). The cells were transfected with 660 ng of pGL4.19 (with or without insert) along with 33 ng Renilla plasmid. Lipofectamine 2000 (Invitrogen, United States) was used for transfection into HEK293 cells, according to the manufacturer’s protocol. Cells were harvested at 48 h after transfection, and luciferase assays were then performed with the Dual-Luciferase Assay Kit (Promega, United States) according to the manufacturer’s instructions. Cell lysates were tested first for firefly luciferase activity and then for Renilla luciferase activity. Firefly luciferase luminescence values were divided by Renilla luciferase luminescence values from the same transfection to control for differences in transfection efficiency.

### Electrophoretic Mobility Shift Assay

Electrophoretic mobility shift assays (EMSA) were conducted using a commercial kit (Viagene Biotech, Co., Changzhou, China) following the manufacturer’s protocol. Nuclear extracts were collected from HEK 293T cells as described previously. We prepared probes for the risk allele G and the non-risk allele A of rs1322330 by annealing 21-bp complementary oligonucleotides and labeling with digoxigenin (DIG)-11-ddUTP (Roche, United States). The DNA/protein binding assay was performed with 10 mg of nuclear extracts using the LightShift Chemiluminescent EMSA Kit (Thermo Fisher Scientific, United States) according to the manufacturer’s recommendations. For competition experiments, nuclear extracts were pre-incubated with excess unlabeled probes. All gel electrophoresis procedures were performed at 4°C. The DNA/protein complexes were detected by streptavidin peroxidase, and signal detection was performed in a 5200 Multi Luminescent Image Analyzer (Tianneng, China).

### Muscle Fiber Type Analysis

Twenty AIS patients and 20 CS patients were randomly selected for the muscle fiber analysis. Immunohistochemical staining was performed to determine the ratio of type I fiber to type II fiber of bilateral paraspinal muscles for each patient. Freshly collected muscles tissues were frozen in isopentane/liquid nitrogen. Embedded samples were cryo-sectioned at a thickness of 10 μm for storage at −80°C until further processing. Muscle fiber type distributions were performed on transverse cryosections (10 μm). Serial sections of each muscle were reacted for adenosine triphosphatase (ATPase) at a pH of 9.4. After reacting with the ATPase, there is a clear differentiation into two fiber types. The type I fibers are more lightly stained and the type II fibers more heavily stained. Slides were photographed at 20X (Zeiss, Lab A1), and the cross-sectional area (CSA) was measured for the two fiber types. Approximately 100 fibers were measured per patients within two to three different fields.

### Purification and Culture of Myosatellite Cells

Paraspinal muscles of five AIS patients and five CS patients were randomly selected for the isolation of myosatellite cells (MSCs). The MSCs were purified as described previously (Laumonier et al., 2017). After purification, the cells were subsequently cultured in a growth medium with DMEM/F-12 complemented with 20% FBS, 1% α-glutamine, and 1% P/S (penicillin–streptomycin) at 37°C in a 5% CO_2_ humidified incubator following the manufacturer’s protocol. Differentiation process was initiated after the cells reached 70%–80% confluence by replacing the growth medium with an equal volume of differentiation medium: DMEM/F-12 complemented with 2% HS, 1% α-glutamine, and 1% P/S. Then, media was changed with a fresh differentiation medium every 2 days. Cell morphology was imaged under the Zeiss Observer A1 system. Cell proliferation assays were performed with a Cell Counting Kit-8 (CCK8). The myogenic differentiation ability was assessed by formation of myotubes, which were immunostained using the myotube-specific *MF20* (myosin heavy chain) antibody. The fusion index of myoblasts was evaluated after 7 days and defined as the percentage of nuclei in *MF20*-stained cells containing two or more nuclei.

### Immunofluorescence Staining

Cells were fixed with 4% paraformaldehyde in phosphate-buffered saline (PBS) followed by permeabilization with 0.1% Triton X-100 in PBS. Then, 5% goat serum in PBS was used for blocking. Cells were then incubated with primary antibody (*MF20*, 1:20, DSHB; *Desmin*, 1:50, Abcam) overnight at 4°C, followed by incubation with secondary antibody (1:500; Jackson ImmunoResearch) at room temperature for 1 h. Nucleus were counterstained with DAPI solution (1:1,000 dilution in PBS) for 10 min on a rocking platform. Images were taken under 100x with the Leica DM5500 system. Images of stained cells were taken using a fluorescence microscope (Observer A1, Zeiss, Germany).

### Transfection of Lentivirus *LBX1*

The MSCs of the control group were transfected with *LBX1* lentivirus or with empty vector. The sh*LBX1*-mCherry lentivirus targeting *LBX1* and sh*Ctrl*-*mCherry* control lentivirus were obtained from GeneCopoeia (Rockville, United States). The transfected cells were cultured in a growth medium containing 1 μg/ml puromycin (Thermo Fisher Scientific) for another 2 days to select the successfully transfected cells. The sequence of sh*LBX1* lentivirus targeting LBX1 was as follows: GAC​ATC​CTC​AAC​AAG​CCG​TCT. Knock-down of LBX1 expression was then validated by real-time quantitative PCR (RT-qPCR). All the transfected cells were cultured until the formation of myotube. Cell viability was then compared among the lentivirus-transfected group, the empty vector group, and the blank group.

### RT-PCR Analysis

Freshly collected muscle tissues were snap frozen immediately in liquid nitrogen and stored at −80°C. The total RNA of muscle biopsies and cells was extracted with TRIzol (Thermo Fisher Scientific, United States) according to the manufacturer’s protocol. Reverse transcription of 2 μg of the total RNA was performed with the High Capacity cDNA Reverse Transcription Kit (Applied Biosystems, CA), followed by qPCR with Power SYBR Green (Thermo Fisher Scientific, United States) using Quantstudio^TM^ 12K Flex real-time PCR platform (Life Technologies, United States). Glyceraldehyde-3-phosphate dehydrogenase (*GAPDH*) was used as the endogenous control gene for the normalization of mRNA expression. The specific primers are as follows: forward 5′-AGG​ACA​TCC​TCA​ACA​AGC​CG-3′, reverse 5′-CAT​ACC​GTC​GCG​GCC​TTC-3′ for the *LBX1* gene; forward 5′-CGG​ACG​TGC​CTT​CTG​AGT​C-3′, reverse 5′-AGC​ACC​TGG​TAT​ATC​GGG​TTG-3′ for the *MyoD* gene; forward 5′-GGG​GAA​AAC​TAC​CTG​CCT​GTC-3′, reverse 5′-AGG​CGC​TCG​ATG​TAC​TGG​AT-3′ for the *MyoG* gene; forward 5′-GGA​GCG​CCA​TCA​GCT​ATA​TTG-3′, reverse 5′-ATC​CGC​ACC​CTC​AAG​ATT​TTC-3′ for the *MYF6* gene; and forward 5′-GAG​TCA​ACG​GAT​TTG​GTC​GT-3′, reverse 5′-TTG​ATT​TTG​GAG​GGA​TCT​CG-3′ for the *GAPDH*. All amplifications were performed in triplicate. The mean value of threshold cycle (Ct) scores was calculated to determine the relative expression level. The expression of the target gene was calculated using ΔΔCt method.

### Protein Extraction and Western Blot

Total protein was extracted from the muscle samples or from cultured cells using the lysing buffer (Invitrogen, CA, United States). Protein quantification was conducted using a BCA Protein Assay Kit (Thermo Scientific, CA, United States) following the manufacturer’s instructions. The protein was subjected to SDS-PAGE and transferred onto polyvinylidene difluoride (PVDF) membranes. Sequentially, the membranes were incubated with rabbit anti-*LBX1* (ab90836, Abcam), rabbit anti-*GAPDH* (ab9485, Abcam), and goat anti-rabbit secondary antibodies (ab205718, Abcam) and visualized *via* enhanced chemiluminescence system. Similarly, rabbit anti-*MyoD1* (13812, Cell Signaling Technology), mouse anti-*MyoG* (F5D, Developmental Studies Hybridoma Bank), and rabbit anti-*MYF6* (11754-1-AP, ProteinTech) were used to test the expression of target genes, respectively*. GAPDH* was used as an internal reference to normalize the quantity of the protein.

### Statistical Analysis

The Hardy–Weinberg equilibrium (HWE) test was performed for both patients and controls. The differences of genotype and allele distributions between patients and controls were calculated using chi-square test. The one-way ANOVA test was used to compare the mRNA expression of *LBX1* among different genotypes of rs1322330. The Student’s *t*-test was used to compare the mRNA expression of *LBX1* between cases and controls. The Pearson correlation analysis was used to determine the relationship between the tissue expression level of *LBX1* and *MyoD*, as well as the relationship between gene expression and the CSA of myotube in AIS patients. All statistical analysis was conducted with SPSS version 19.0 (SPSS Inc., Chicago, United States). A *p* value of <0.05 was considered statistically significant.

## Results

### Demographic Data

For the genetic association analysis, the mean age was 15.4 ± 3.7 years (range, 11–18 years) for the patients and 19.1 ± 2.9 years (range, 17–22 years) for the healthy controls. The mean Cobb angle was 35.7° ± 15.2°, ranging from 20° to 72°. For the tissue expression analysis, the mean age was 14.4 ± 2.1 years for AIS patients and 14.3 ± 2.3 years for CS patients (*p* = 0.82), respectively. For CS patients, 20 had hemivertebra and the other eight were diagnosed as malformation of vertebral body. The mean Cobb angle was 55.1° ± 8.4° for AIS and 57.2° ± 7.7° for CS (*p* = 0.22), respectively. The proximal vertebral region for sample collection ranged from T3 to T5 level of the thoracic region (Supplementary Table S1).

### Fine-Mapping of the Putative Regulatory Region

**FIGURE 1:**
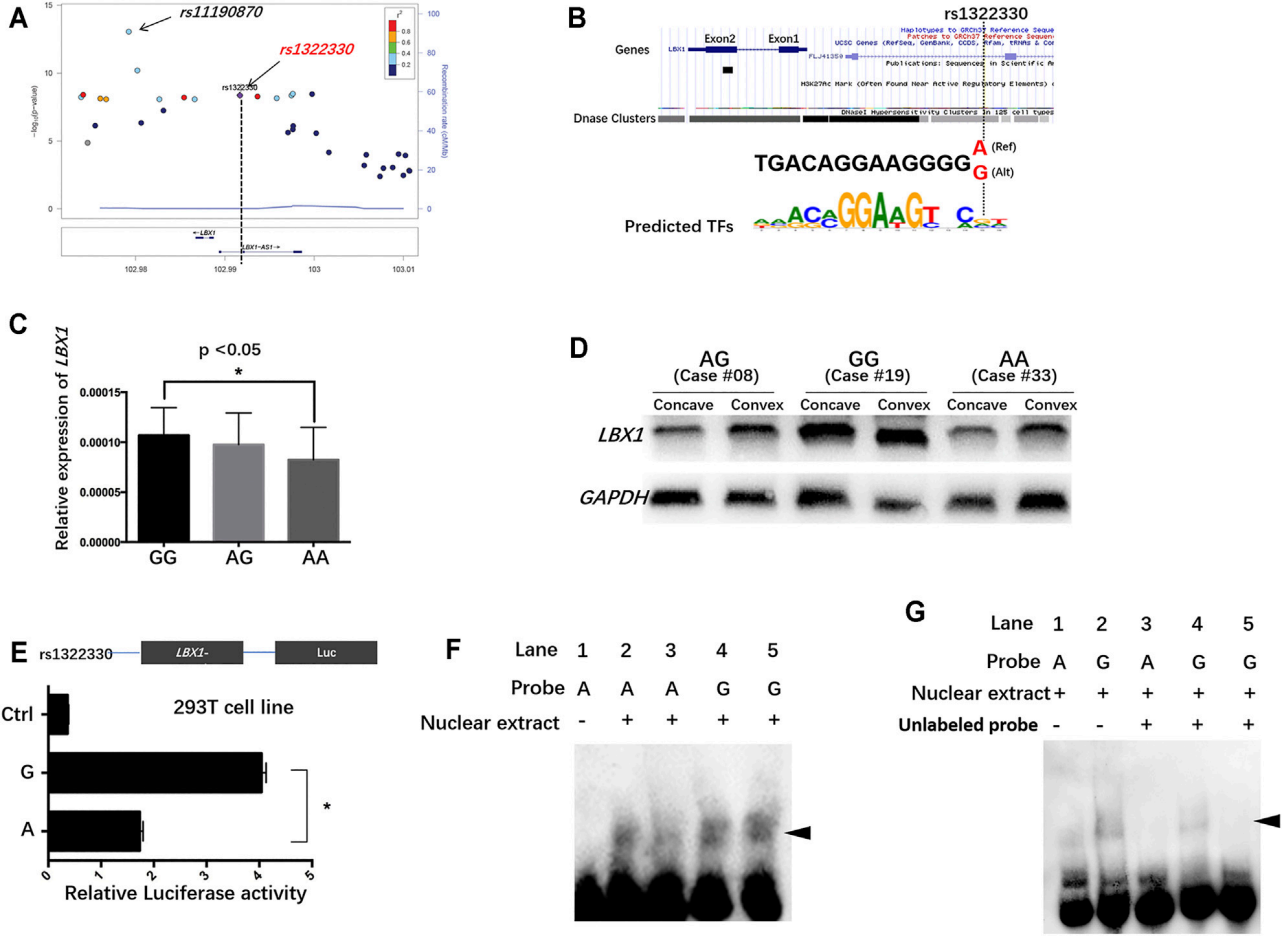
The regulatory mechanism underlying *LBX1* expression in the paraspinal muscles of adolescent idiopathic scoliosis (AIS). **(A)** Fine-mapping of the previously reported susceptible loci showed that rs1322330, located in the promoter region of *LBX1*, was remarkably associated with AIS with genome-wide significance (*p* = 4.25 × 10^−9^). **(B)** Genome browser of rs1322330 indicated altered activity of DNase at this locus. The variant A/G is predicted to alter the binding affinity of certain transcriptional factors. **(C)** Patients with genotype AA of rs1322330 were found to have remarkably decreased *LBX1* mRNA expression as compared with those with genotype GG. **(D)** WB analysis confirmed that genotype AA was indicative of remarkably lower *LBX1* protein expression in paraspinal muscles. **(E)** Luciferase reporter assays for rs1322330 (A/G) on *LBX1* promoter activity were performed in the HEK293 cell lines. Empty vector pGL4.19-basic vector was used as reference. Luciferase assay showed that allele A of rs1322330 can lead to nearly 50% decrease of the transcriptional activity of *LBX1* promoter as compared with allele G. **(F)** Electrophoretic mobility shift assay (EMSA) showed that Mut-Oligos **(A)** had a lower ability of transcription factor combination as compared with Wt-Oligos **(G)**. **(G)** The addition of competitive unlabelled probe in lines 3–5 led to decreased formation of DNA–protein complexes.

The imputation analysis indicated that rs1322330 was the most significant SNP located in the promoter region of *LBX1* (*p* = 4.25 × 10^−9^) (Figure 1A; Supplementary Table S2). As shown in Table 1, the independent replication analysis confirmed that the frequency of allele A was significantly higher in the patients than in the controls (0.676 vs. 0.618, *p* = 1.42 × 10^−4^). A combination of the imputation analysis and the replication analysis showed that rs1322330 was associated with AIS with genome-wide significance (*p* = 6.08 × 10^−14^). Allele A can significantly add to the risk of AIS with an odds ratio (OR) of 1.42 (95% confidential interval: 1.03–1.55).

**TABLE 1:** The association of rs1322330 with the development of AIS.

Stage	Sample size		RAF		*p*	OR (95% CI)
	Cases	Controls	Cases	Control		
Imputation	980	1,499	0.683	0.585	4.25 × 10^–9^	1.53 (1.35–1.72)
Replication	1,000	1,000	0.676	0.618	1.42 × 10^–4^	1.29 (1.13–1.47)
Combined	1,980	2,499	0.679	0.599	6.08 × 10^–14^	1.42 (1.03–1.55)

RAF, risk allele frequency; OR, odds ratio; CI, confidential interval.

### Regulatory Effect of rs1322330 on the Expression Level of *LBX1*

AIS patients with genotype AA were found to have significantly lower expression of the *LBX1* as compared with those with genotype GG (Figure 1C, D). The online databases (HaploReg and UCSC Genome Browser) indicated that rs1322330 was located in a potential TF-binding region with altered signal of DNase cluster (Figure 1B; Kent et al., 2002; Ward and Kellis, 2012). To further address the functional role of rs1322330, reporter gene constructs containing the risk allele (allele A) and non-risk allele (allele G) were prepared. The insertion of the *LBX1* promoter fragment consistently resulted in an augmentation of the luciferase activity as compared with the blank construct. The *LBX1*/A-Luc construct had significantly lower luciferase activity than the *LBX1*/G-Luc construct (Figure 1E).

### Electrophoretic Mobility Shift Assay

The shift band corresponding to allele G probe–protein complexes (Figure 1F, lines 4 and 5) was significantly more intense than that corresponding to allele A probe–protein complexes (Figure 1F, lines 2 and 3), thus suggesting that the two alleles had different affinities for certain transcription factor within the nuclear extracts. A 50-fold excess of unlabeled probes remarkably abrogated the formation of DNA–protein complexes (Figure 1G, lines 3–5), thus confirming the specificity of these interactions.

### The Relationship Between *LBX1* Expression and Distribution of Muscle Fiber Type

**FIGURE 2:**
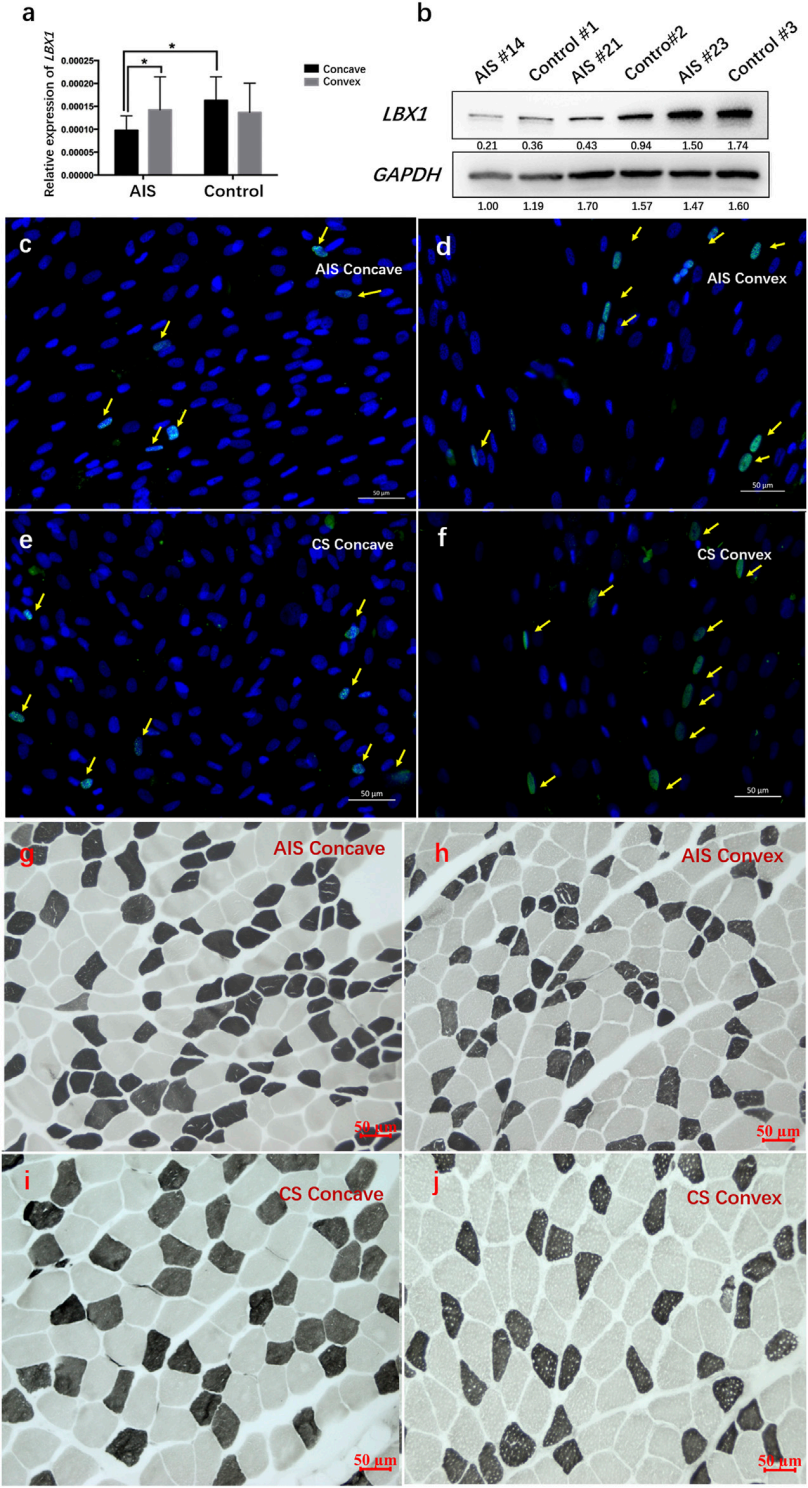
AIS patients had globally down-regulated *LBX1* expression as well as smaller muscle fiber in the bilateral sides of paraspinal muscles. **(A)** The mRNA expression of *LBX1* was remarkably deceased in the bilateral side of AIS as compared with that in the bilateral sides of CS patients. **(B)** The protein expression of *LBX1* was remarkably lower in the paraspinal muscles of AIS than CS. **(C–F)** Target protein was stained in green (arrows). DAPI was stained in blue. Remarkably lesser expression of *LBX1* was found in the paraspinal muscles of AIS as compared with CS. **(G–J)** ATPase staining following preincubation at pH 9.4 showed the lightly stained type I fibers and the darkly stained type II fibers. Globally smaller type I fiber in the paraspinal muscles of AIS can be observed as compared with CS patients.

RT-PCR results are summarized in Supplementary Table S3. AIS patients were found to have remarkably lower mRNA expression of *LBX1* in the concave side than in the convex side (Figure 2A). For CS patients, there was no significant difference regarding mRNA expression between the concave side and the convex side. The average mRNA expression of *LBX1* was remarkably lower in AIS patients than in CS patients (0.00024 ± 0.000089 vs. 0.00030 ± 0.00012, *p* = 0.03). Both WB and immunofluorescence analysis confirmed that the protein expression of *LBX1* was remarkably lower in AIS muscles than in CS muscles (Figure 2B–F; Supplementary Figure S1).

As shown in Figure 2, there was significantly less type I fiber in the concave muscles as compared with the convex muscles in AIS patients (44.4% ± 12.7% vs. 65.4% ± 16.3%, *p* = 0.004). By contrast, there was no significant difference regarding the proportion of fiber type between the bilateral sides of paraspinal muscles in CS patients (67.4% ± 21.2% vs. 79.5% ± 25.2%, *p* = 0.26) (Supplementary Table S4). Intergroup comparison showed that there was globally less type I fiber in the bilateral paraspinal muscles of AIS patients than in CS patients (55.2% ± 14.1% vs. 73.5% ± 22.4%, *p* = 0.04). In addition, the average CSA of muscle fibers was remarkably smaller in AIS than that in CS (1,350.3 ± 256.1 vs. 1,712.3 ± 374.4 μm^2^, *p* = 0.02) (Supplementary Table S4). The correlation analysis showed that the mRNA expression of *LBX1* was remarkably correlated with the CSA of muscle fibers in the paraspinal muscles of AIS patients (*r* = 0.375, *p* = 0.04).

### The Influence of *LBX1*-Knowndown on the Biological Features of MSCs

**FIGURE 3:**
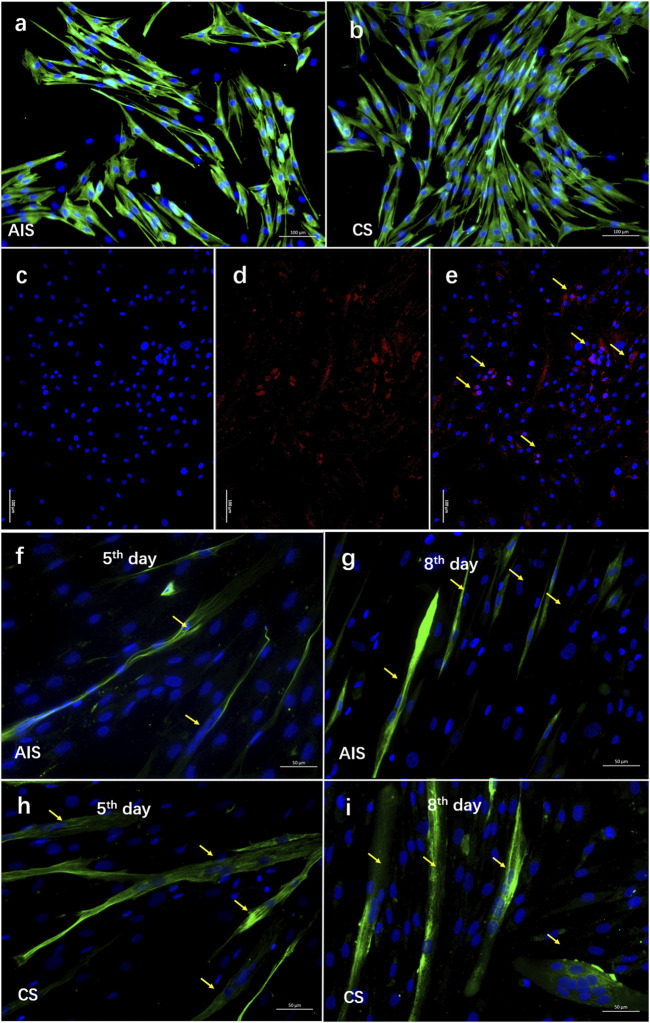
A decreased differentiation ability was observed in myosatellite cells isolated from AIS. **(A–E)** The myosatellite cells (MSCs) were isolated and purified from the paraspinal muscles of five AIS patients and five CS patients. Desmin (the muscle-specific intermediate filament) was stained in green. *PAX7* was stained in red (arrows). DAPI was stained in blue. A pure population of MSCs (>90%) was confirmed *via* the immunostaining of *Desmin* and *Pax7*. (F–I) At the fifth day and eighth day of MSC culture, formation of myotube was observed for both groups (arrows). Compared with the CS MSCs, remarkably smaller myotube was observed in AIS MSCs.

The MSCs were confirmed *via* the immunostaining of *Desmin* and *Pax7* (Figure 3A–E). The CCK8 assay showed that MSCs isolated from the AIS concave muscles had obviously lower viability than those of CS patients (Supplementary Figure S2A; Supplementary Table S5). Moreover, compared with the CS group, a remarkably lower fusion index of the myotube was observed for the AIS group at the fifth day and the eighth day, respectively (Figure 3F–I; Supplementary Table S6).

**FIGURE 4:**
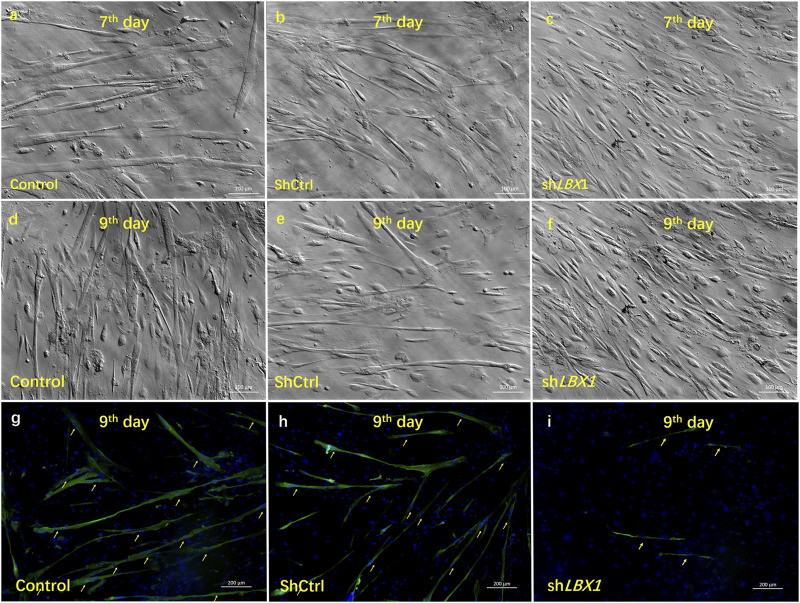
The influence of silencing *LBX1* on the viability of MSCs. **(A–F)** For MSCs isolated from CS, *LBX1* was knocked down *via* lentivirus transfection. Compared with the blank group and the *ShCtrl* group, decreased cell viability could be observed in the *ShLBX1* group at the seventh day and the ninth day after MSC culturing. (G–H) *MF20* was stained in red. DAPI was stained in blue. Immunofluorescence staining showed remarkably fewer and smaller myotube (arrows) in the Sh*LBX1* group at the ninth day of MSC culturing.

To further clarify the involvement of *LBX1* in skeletal MSC proliferation and differentiation, we inhibited *LBX1* in the MSCs of CS patients with lentivirus, which effectively resulted in the knockdown of *LBX 1* by more than 80% (Supplementary Figure S3). A significantly inhibited proliferation rate was observed in the *ShLBX1* group by the CCK-8 test (Figure 4; Supplementary Figure S2B; Supplementary Table S7). Moreover, a remarkably decreased formation of the myotube was observed after the knockdown of *LBX1* (Figure 4; Supplementary Figure S4).

**FIGURE 5:**
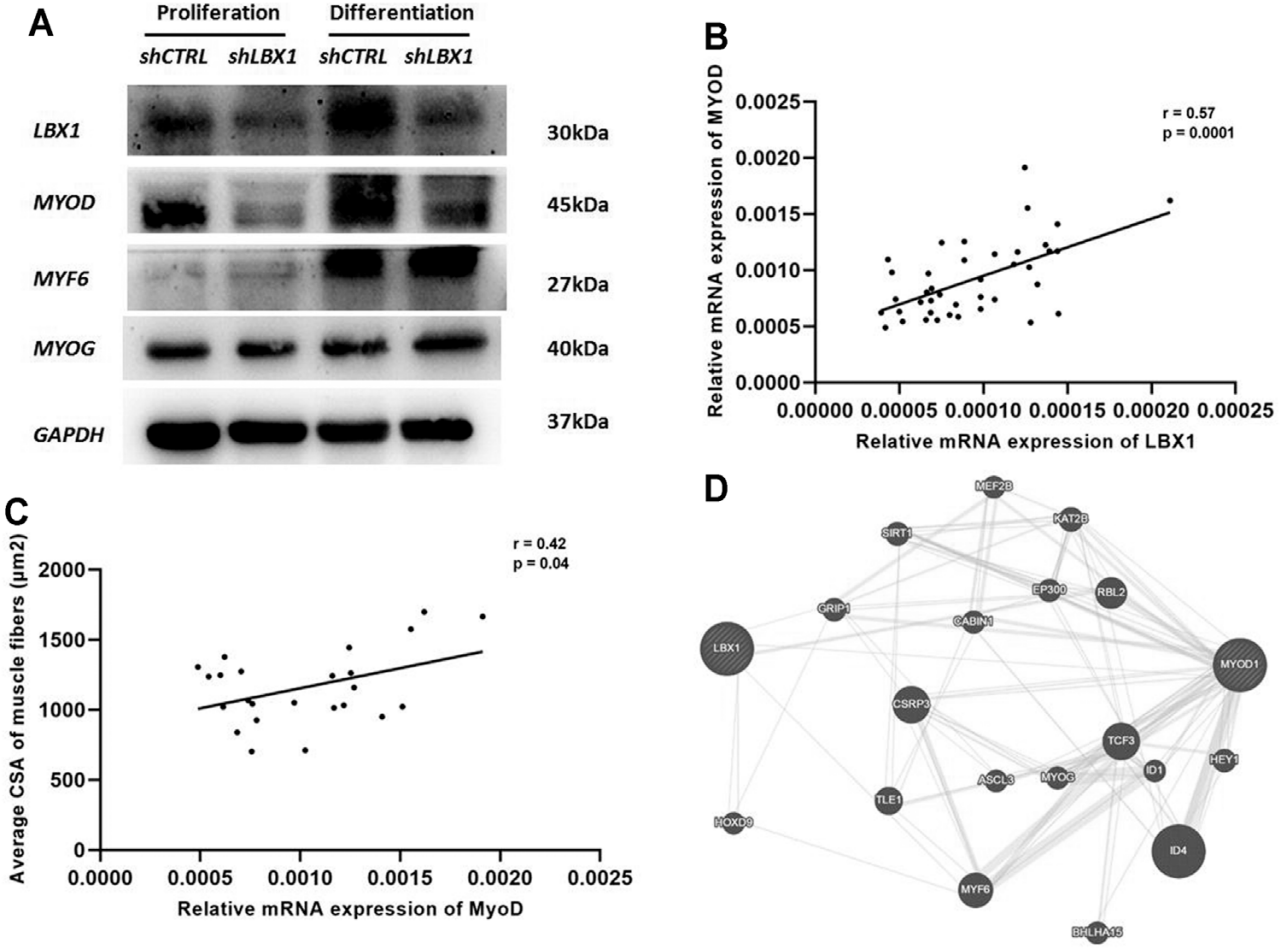
*MyoD* was involved in the regulation of *LBX1* on myogenesis in AIS. **(A)** Protein expression level of myogenic markers in MSCs with *LBX1* knockdown was validated by western blot at proliferation and differentiation stages. *GAPDH* was used as internal control. For both proliferation and differentiation stages, a remarkably decreased protein expression of *MyoD* was confirmed in the *LBX1*-lentivirus-transfected cells. **(B)** Tissue expression analysis in 48 AIS patients showed that the mRNA expression of *LBX1* was positively correlated with the expression of *MyoD* (*r* = 0.57, *p* < 0.01). **(C)** The cross-sectional area (CSA) of myotube and the expression of *MyoD* were evaluated at the ninth day after MSC culture. The mRNA expression of *MyoD* was significantly correlated with the CSA of the type I fiber (*r* = 0.42, *p* = 0.04). **(D)** Functional pathway enrichment of differential genes was analyzed based on KEGG and BIOCARTA pathway databases. Here, we showed that there was a lack of direct interaction between *LBX1* and *MyoD*.

As the member of myogenesis regulatory family, the expression of *MyoG*, *MyoD*, and *MYF6* was analyzed at both proliferation and differentiation stages of MSCs. The expression of *MyoD* was significantly decreased in the *ShLBX1* group as compared with the control group (Figure 5A). As for the expression of the other two genes, no significant difference was found between the two groups (Figure 5A). As shown in Supplementary Table S3, the mean expression of *MyoD* was remarkably lower in the paraspinal muscles of AIS than CS (0.00237 ± 0.00092 vs. 0.00294 ± 0.00129, *p* = 0.03). There was a significant correlation between the mRNA expression of *LBX1* and *MyoD* (*r* = 0.57, *p* = 0.0001) (Figure 5B; Supplementary Table S8). In addition, the expression of *MyoD* in paraspinal muscles of AIS patients was remarkably correlated with the mean CSA of muscle fibers (*r* = 0.42, *p* = 0.04) (Figure 5C).

## Discussion

Previous GWASs of AIS have uncovered several novel susceptible genes such as *LBX1*, *GPR126*, *BCL2*, and *BNC2* (Jiang et al., 2013; Kou et al., 2013; Ogura et al., 2015; Zhu et al., 2015). Uniquely, the association between rs11190870 of *LBX1* and AIS was supported by replication studies in different populations (Jiang et al., 2013; Nada et al., 2018). Through fine-mapping of a 40-kb region surrounding rs11190870, we pinpointed a potentially functional SNP rs1322330 located in the promoter region of *LBX1*. Based on a large independent cohort of patients and controls, we further confirmed that rs1322330 was remarkably associated with the development of Chinese AIS. Allele A of rs1322330 was found to remarkably add to the risk of AIS with genome-wide significance.

The role of rs1322330 in the regulation of *LBX1* expression remains obscure in patients with AIS. For the first time, we analyzed the tissue expression of *LBX1* in patients with different genotypes of rs1322330. Patients with genotype AA were observed to have significantly decreased expression of *LBX1* as compared with those with genotype GG. Through luciferase reporter assays, we found that the promoter construct with the A nucleotide had remarkably less promoter activity than the G nucleotide construct. These findings were consistent with the genotyping analysis of rs1322330, which showed that patients had obviously higher frequency of genotype AA than normal controls. As previously reported, AIS patients were found to have significantly deceased expression of *LBX1* in paraspinal muscles (Xu et al., 2020).Taken together, rs1322330 may play an important role in the regulation of *LBX1* expression in AIS tissues.

It has been well documented that functional variants in the promoter region can alter the binding affinity of certain TFs and thus influence the promoter activity (Saito et al., 2001; Wang et al., 2010). To substantiate the potential regulatory role of rs1322330, the binding ability of probes containing allele A or G with the nuclear extracts was assessed by EMSA. The probe containing allele A was observed to have less binding affinity as compared to the probe containing allele G. Consistently, unlabeled competition oligonucleotide probes dramatically eliminated specific binding in the cell nuclear extracts. To sum up, these findings suggested that the rs1322330 may regulate the promoter activity of the *LBX1*. In future studies, chromatin immunoprecipitation experiment is warranted to further reveal the underlying regulatory elements of rs1322330.

To further unveil the functional role of *LBX1* in the etiology of AIS, the fiber composition of paraspinal muscles was investigated for both AIS and CS patients. A remarkably smaller CSA of muscle fibers was found in AIS as compared with CS. Interestingly, *LBX1* expression was found to be correlated with the CSA of the muscle fibers. To exclude the influence of mechanical stress on the differentiation of muscle fibers, we isolated and purified MSCs from the proximal paraspinal muscles of both AIS and CS patients. MSCs of the AIS group presented significantly lower viability as compared with those of the CS group. After the formation of myotubes, remarkably less fusion index was observed in the AIS group. Based on these findings, it was hereby worthwhile to further uncover the relationship between *LBX1* and abnormal muscle fiber formation in AIS.

To date, limited knowledge concerning the biological role of *LBX1* in the myogenesis of AIS has been reported (Fernandez-Jaen et al., 2014). For the first time, we silenced *LBX1* expression in the MSCs using lentivirus and evaluated its influence on the cell proliferation and differentiation. Knockdown of *LBX1* resulted in significantly inhibited cell viability and decreased myotube formation. Interestingly, *MyoD* expression was also down-regulated in *LBX1*-silenced MSCs. As a key regulator affecting the differentiation of muscle fibers (Hennebry et al., 2009), the expression of *MyoD* was found to be correlated with that of *LBX1* in both AIS and CS tissues. As reported in earlier literatures, *LBX1* and *MyoD* were both involved in the *Wnt/B-catenin* pathway that could regulate myogenesis (Watanabe et al., 2007). Taken together, we therefore speculated that *LBX1* may be involved in the differentiation of myoblast and subsequent myotube formation *via* the regulation of *MyoD*. The underlying mechanism needs more investigation in future study.

As reported in earlier literatures, *MyoD* is involved in the regulation of the shift of muscle fiber type composition (Hennebry et al., 2009; Tee et al., 2009). Different functions of type I and type II fibers have been reported in previous studies (Feng et al., 2011; Huang et al., 2012; Hopker et al., 2013). Theoretically, slow-twitch fibers (type I) in the paraspinal muscles may be more responsible for the static control of the truck as compared with the fast-twitch fibers (type II). Interestingly, it has been well documented that AIS patients had reduced hand grip strength as well as weakened body balance (Le Berre et al., 2017; Sahin et al., 2019). It was thus probable that dysregulated myogenesis in the paraspinal muscle can make patients more vulnerable to the development or progression of the spinal curvature. In future studies, establishment of animal models is warranted to determine the role of myogenic factors in the etiology of AIS.

Two limitations of the present study should be addressed. First, there were no age-matched normal controls included in the expression analysis. It is extremely difficult to collect paraspinal muscles from age-matched non-scoliosis children. Patients undergoing spine surgery due to trauma may be qualified as a normal control, which, however, was rarely accoutered in clinical practice. Second, although we proved that rs1322330 could influence the transcriptional activity of *LBX1* promoter, more evidence is needed to prove the binding of certain transcriptional factor at this site. In future studies, a more functional assay could be used to further reveal the regulatory mechanism of rs1322330.

To conclude, SNP rs1322330 is functionally associated with the development of AIS in Chinese Han population. Allele A of rs1322330 may affect the promoter activity of the *LBX1*. *LBX1* may be involved in the etiology of AIS through involvement in the myogenesis of paraspinal muscles. Further functional analysis is warranted to determine the downstream pathway of *LBX1* that contributes to the abnormal myogenesis of AIS.

## Data Availability

The data presented in the study are deposited in the Figshare repository, accession number: https://doi.org/10.6084/m9.figshare.16864672.
