# Supplementary material for: A Functional SNP in the Promoter of LBX1 Is Associated With the Development of Adolescent Idiopathic Scoliosis Through Involvement in the Myogenesis of Paraspinal Muscles
Source: Front Cell Dev Biol. 2021 Nov 30;9:777890. doi: 10.3389/fcell.2021.777890 (PMC8670502; doi:10.3389/fcell.2021.777890)
Supplement: Supplementary file 1 [file DataSheet1.docx]

**Supplementary figures**

**Supplementary figure 1 The expression of *LBX1* in MSCs and paraspinal muscles**


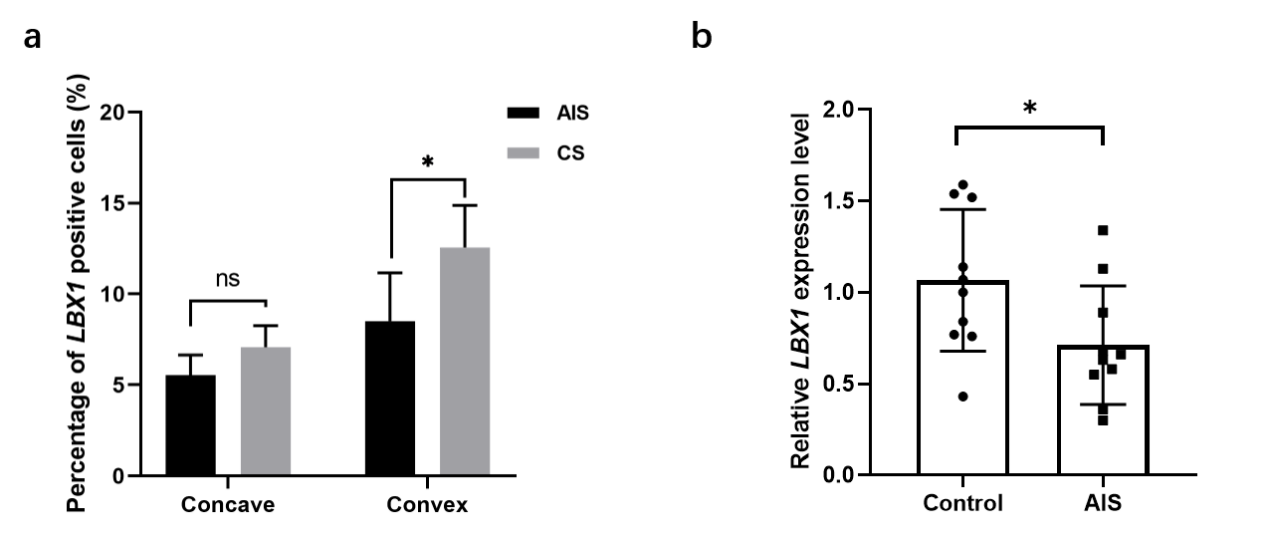
 **a.** The percentage of *LBX1* positive cells from the convex side of AIS patients (n=5) was significantly lower than that of CS patients (n=5). No remarkably difference was observed between the concave sides of AIS and CS patients. **b**. The protein expression level of *LBX1* was significantly lower in the paraspinal muscles of AIS patients (n=10) than that of the CS patients (n=10). Data are shown as mean ± SD. *P<0.01; ns, not significant.

**Supplementary figure 2 Viability tests of the MSCs cells**

**
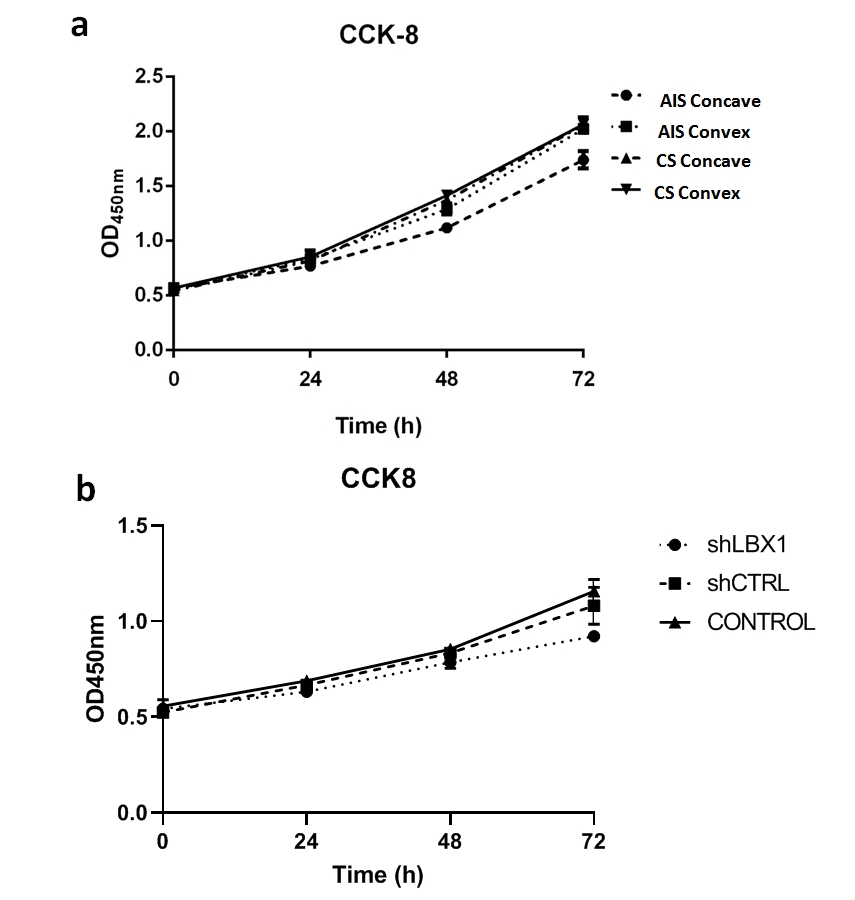
**

**a.** MSCs were isolated and purified from 5 AIS patients and 5 CS patients. The proliferation rate of MSCs was compared between the AIS group and the CS group. MSCs in the AIS group had remarkably lower proliferation rate than those in the CS group. Moreover, MSCs cells isolated from the AIS concave muscles had lower proliferation rate than those isolated from the convex muscles. There was no significant difference between the bilateral sides of CS group regarding the MSCs proliferation rate. **b.** MSCs of CS patients were transfected with lentivirus *LBX1* or with empty vector. MSCs in the *ShLBX1* had remarkably lower proliferation rate as compared with the empty vector or with the blank group.

**Supplementary figure 3 The transfection efficiency of lentivirus**

**
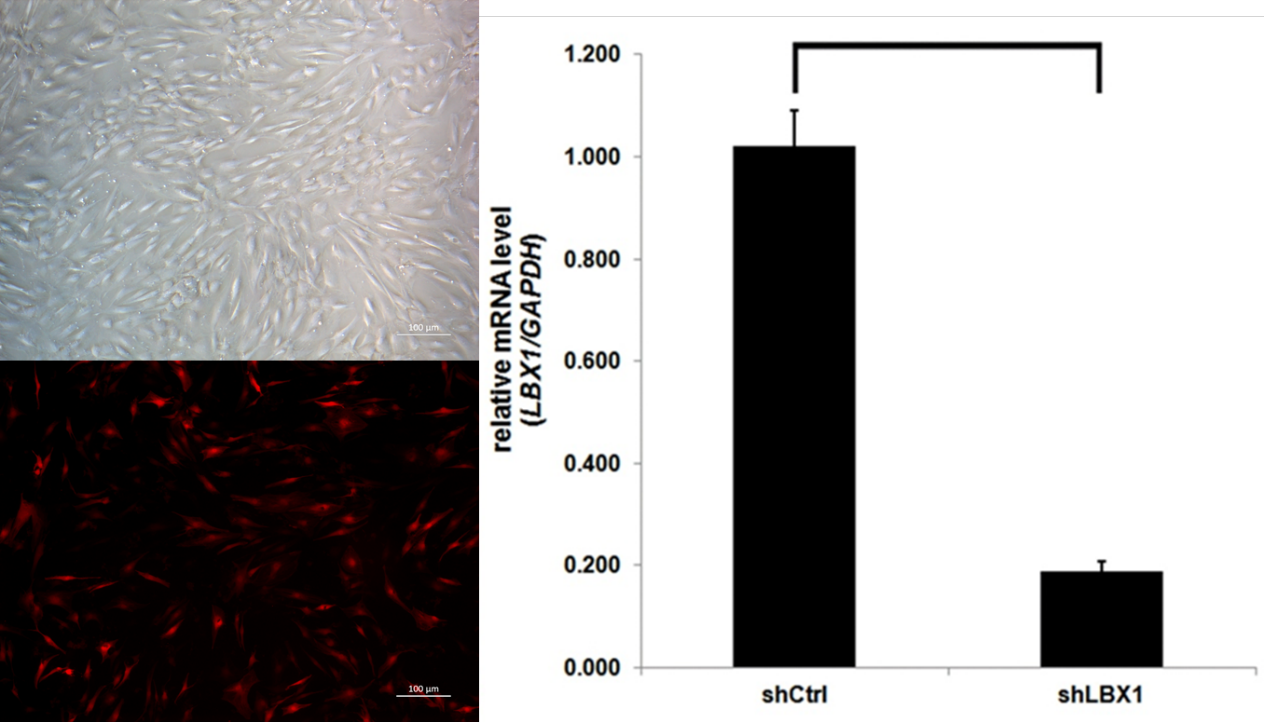
**

The immunostaining confirmed successful transfection of lentivirus *LBX1*. The expression of mCherry signal (red) was detected with fluorescence microscope (Leica DMIRB system) to verify the efficiency of transfection. The mRNA expression of *LBX1* was remarkably decreased in the *ShLBX1* group as compared with the *ShCtrl* group. >80% knockdown of *LBX1* was observed after the transfection of lentivirus *LBX1*.

**Supplementary figure 4 Influence of *LBX1* knockdown on the viability of MSCs**

**
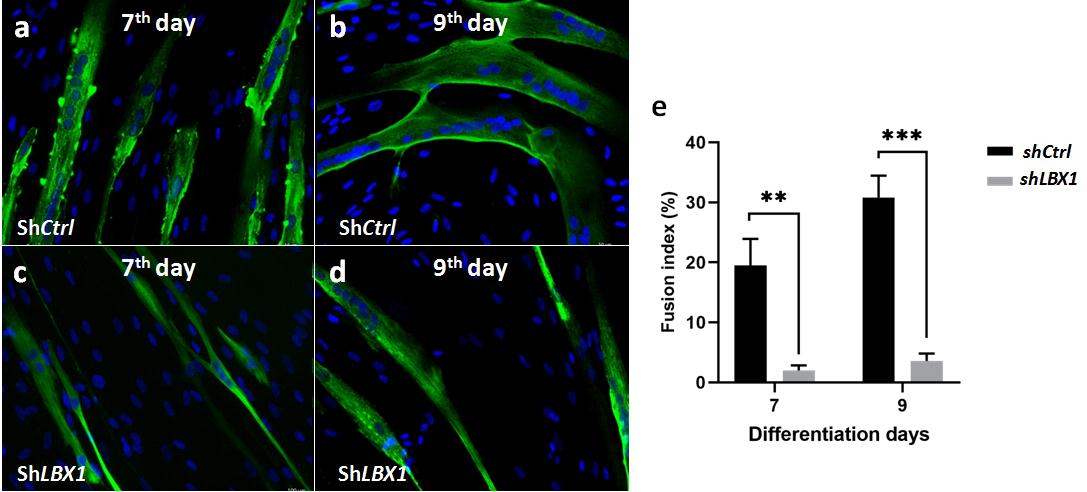
**

**a-d** Immunoﬂuorescence staining of the MSCs was performed at the 7th day and the 9th day after the transfection of lentivirus. *MF20* was stained in green. DAPI was stained in blue. Remarkably decreased number of myotube was observed after the knockdown of *LBX1*. **e.** Primary MSCs transduced with *shLBX1* demonstrated significantly lower fusion index at the 7th day and the 9th day after differentiation (19.5% ± 4.37% vs. 2.01% ± 0.84%, p < 0.01 for the 7th day; 30.83% ± 3.61% vs. 3.57% ± 1.23%, p <0.01 for the 9th day). (n = 3 experimental replicates, Values are represented as mean ± SEM. ***P<0.05, 2-tailed unpaired Student’s t test.).

**Supplementary Tables**

**Supplementary Table 1 Demographic data of the subjects included in the tissue analysis**

|  | AIS  (n = 134) | CS  (n = 20) | p |
| --- | --- | --- | --- |
| Age (yrs) | 14.4 ± 2.1 | 14.3 ± 2.3 | 0.82 |
| Cobb angle (degrees) | 55.1 ± 8.4 | 57.2 ± 7.7 | 0.22 |
| Body mass index (kg/m^2^) | 17.6 ± 3.2 | 18.1 ± 4.6 | 0.57 |
| Location of tissue collection | T3 - T5 | T3 - T5 | N/A |

Baseline characteristics of the subjects were summarized. The two groups were matched in terms of age, body mass index and curve magnitude.

**Supplementary Table 2 Imputation results of putative regulatory variants**

| **SNP** | **BP** | **Ref Allele** | **MAF** | | **OR** | **P** |
| --- | --- | --- | --- | --- | --- | --- |
|  |  |  | **Patients** | **Controls** |  |  |
| rs11190870 | 102979207 | C | 0.369 | 0.476 | 0.6293 | 8.68 x 10^-14^ |
| rs11598177 | 102980156 | T | 0.527 | 0.437 | 1.461 | 6.35 x 10^-11^ |
| rs678741 | 102997581 | A | 0.533 | 0.445 | 1.436 | 3.28 x 10^-9^ |
| rs10883597 | 102999754 | C | 0.526 | 0.438 | 1.429 | 3.45 x 10^-9^ |
| rs3950032 | 102974109 | C | 0.323 | 0.415 | 0.6573 | 3.83 x 10^-9^ |
| **rs1322330** | **102991659** | **G** | **0.317** | **0.415** | **0.6449** | **4.25 x 10^-9^** |
| rs679206 | 102997450 | T | 0.533 | 0.445 | 1.436 | 4.43 x 10^-9^ |
| rs625039 | 102993649 | A | 0.315 | 0.412 | 0.6466 | 5.04 x 10^-9^ |
| rs1535462 | 102973872 | A | 0.523 | 0.440 | 1.425 | 5.95 x 10^-9^ |
| rs1407409 | 102985407 | C | 0.319 | 0.417 | 0.6438 | 6.25 x 10^-9^ |
| rs594791 | 102995796 | T | 0.365 | 0.470 | 0.6347 | 7.09 x 10^-9^ |
| rs76319884 | 102976006 | G | 0.321 | 0.419 | 0.6392 | 7.20 x 10^-9^ |
| rs1322331 | 102986589 | A | 0.530 | 0.441 | 1.445 | 8.00 x 1^0-9^ |
| rs1322332 | 102982648 | T | 0.524 | 0.436 | 1.451 | 8.31 x 10^-9^ |
| rs7914775 | 102976661 | T | 0.321 | 0.419 | 0.6418 | 8.40 x 10^-9^ |
| rs79648198 | 102983088 | T | 0.285 | 0.371 | 0.6477 | 5.50 x 10^-8^ |
| rs74152815 | 102980608 | G | 0.076 | 0.041 | 1.894 | 4.62 x 10^-7^ |
| rs74152812 | 102975446 | A | 0.079 | 0.044 | 1.846 | 7.17 x 10^-7^ |
| rs45561036 | 102997627 | A | 0.067 | 0.036 | 1.949 | 7.66 x 10^-7^ |
| rs60208745 | 102997603 | C | 0.067 | 0.036 | 1.912 | 1.39 x 10^-6^ |
| rs56142203 | 102997054 | G | 0.068 | 0.037 | 1.876 | 2.32 x 10^-6^ |
| rs7919667 | 102968878 | T | 0.394 | 0.329 | 1.342 | 2.48 x 10^-6^ |
| rs74152820 | 103000216 | C | 0.065 | 0.036 | 1.902 | 2.55 x 10^-6^ |
| rs145555152 | 103015564 | T | 0.101 | 0.065 | 1.615 | 8.24 x 10^-6^ |
| rs79263639 | 102974606 | A | 0.078 | 0.048 | 1.7 | 1.30 x 10^-5^ |
| rs140149201 | 103015544 | T | 0.101 | 0.067 | 1.56 | 3.00 x 10^-5^ |
| rs66976047 | 103001619 | T | 0.460 | 0.513 | 0.7721 | 6.66 x 10^-5^ |
| rs74152811 | 102970053 | A | 0.063 | 0.038 | 1.709 | 7.06 x 10^-5^ |
| rs17113827 | 103009461 | T | 0.465 | 0.517 | 0.7782 | 9.02 x 10^-5^ |
| rs55776128 | 103005818 | T | 0.465 | 0.517 | 0.778 | 9.93 x 10^-5^ |
| rs56329913 | 103010232 | C | 0.464 | 0.516 | 0.7806 | 0.000113 |
| rs6584419 | 102968882 | C | 0.327 | 0.281 | 1.243 | 0.000613 |
| rs11190876 | 103005582 | G | 0.399 | 0.351 | 1.231 | 0.000625 |
| rs1885517 | 103008802 | G | 0.400 | 0.353 | 1.222 | 0.000889 |
| rs12259511 | 103007748 | T | 0.399 | 0.353 | 1.22 | 0.000995 |
| rs56116649 | 103014469 | A | 0.101 | 0.074 | 1.388 | 0.001438 |
| rs10883598 | 103010619 | T | 0.400 | 0.355 | 1.212 | 0.001467 |
| rs10883599 | 103010664 | A | 0.400 | 0.355 | 1.21 | 0.001586 |
| rs11190882 | 103014632 | G | 0.400 | 0.356 | 1.207 | 0.001853 |
| rs12774109 | 103013461 | G | 0.400 | 0.356 | 1.207 | 0.001853 |
| rs12263774 | 103009986 | C | 0.101 | 0.076 | 1.35 | 0.003387 |
| rs66480325 | 103014673 | A | 0.464 | 0.501 | 0.8299 | 0.004198 |
| rs116457491 | 103015357 | C | 0.437 | 0.408 | 1.218 | 0.01028 |
| rs941910 | 102969972 | T | 0.267 | 0.235 | 1.185 | 0.01093 |
| rs10786627 | 102971968 | A | 0.268 | 0.236 | 1.182 | 0.01226 |
| rs7913672 | 102972695 | T | 0.267 | 0.236 | 1.179 | 0.01375 |
| rs12258229 | 102980899 | A | 0.006 | 0.002 | 3.463 | 0.02165 |
| rs12356729 | 103012690 | A | 0.032 | 0.044 | 0.6969 | 0.02444 |
| rs74152810 | 102966987 | T | 0.031 | 0.021 | 1.489 | 0.02785 |
| rs72843308 | 103014692 | T | 0.462 | 0.490 | 0.8678 | 0.03237 |
| rs78697882 | 103002063 | A | 0.433 | 0.459 | 0.8614 | 0.03401 |
| rs11190883 | 103019352 | A | 0.449 | 0.477 | 0.8861 | 0.04671 |
| rs12359268 | 103009594 | C | 0.032 | 0.041 | 0.7656 | 0.09783 |
| rs11190878 | 103009908 | G | 0.032 | 0.041 | 0.7724 | 0.1099 |
| rs55876166 | 103007660 | T | 0.138 | 0.153 | 0.878 | 0.1359 |
| rs12778405 | 102966414 | T | 0.134 | 0.120 | 1.144 | 0.1364 |
| rs56186033 | 103007661 | A | 0.136 | 0.151 | 0.8807 | 0.1466 |
| rs11190877 | 103006244 | G | 0.026 | 0.033 | 0.7855 | 0.1772 |
| rs11190881 | 103011477 | T | 0.133 | 0.120 | 1.126 | 0.1887 |
| rs35999315 | 102979555 | T | 0.021 | 0.027 | 0.781 | 0.208 |
| rs941909 | 102988345 | A | 0.023 | 0.028 | 0.8092 | 0.2672 |
| rs12778495 | 102974840 | T | 0.022 | 0.027 | 0.8209 | 0.3075 |
| rs34835363 | 102991696 | T | 0.151 | 0.140 | 1.088 | 0.3083 |
| rs55822730 | 103017246 | G | 0.445 | 0.434 | 1.082 | 0.3245 |
| rs56382812 | 103017241 | T | 0.451 | 0.461 | 0.925 | 0.3368 |
| rs10786628 | 102982697 | C | 0.151 | 0.142 | 1.074 | 0.3847 |
| rs7924030 | 102969899 | G | 0.130 | 0.123 | 1.073 | 0.4329 |
| rs7893223 | 102970161 | C | 0.020 | 0.018 | 1.155 | 0.5022 |
| rs11593547 | 103001055 | T | 0.030 | 0.033 | 0.9024 | 0.5503 |
| rs66765139 | 103015827 | G | 0.451 | 0.445 | 1.043 | 0.6022 |
| rs12773591 | 102999019 | G | 0.028 | 0.026 | 1.071 | 0.7044 |
| rs111288279 | 103015510 | G | 0.457 | 0.454 | 1.019 | 0.7998 |
| rs11190873 | 102996764 | A | 0.021 | 0.020 | 1.043 | 0.8408 |
| rs28594372 | 102973890 | A | 0.015 | 0.016 | 0.9623 | 0.8728 |
| rs11190872 | 102996362 | T | 0.024 | 0.024 | 1.02 | 0.9169 |
| rs4917933 | 102992732 | A | 0.001 | 0.001 | 1.041 | 0.9649 |
| rs12253855 | 102971520 | G | 0.022 | 0.022 | 1.002 | 0.9911 |

Through imputaion analysis, the fine-mapping results of a 40 kb region around rs11190870 was summarized. As rs1322330 was the only significant SNP that was located in the promoter region of LBX1, it was further replicated for the eligibility to be investigated for functional role.

**Supplementary Table 3 Gene expression in the bilateral paraspinal muscle of the AIS and CS patients**

|  | AIS (n = 48) | | |  | Congenital scoliosis (n = 24) | |  |
| --- | --- | --- | --- | --- | --- | --- | --- |
|  | Concave side | | Convex side | p | Concave side | Convex side | p |
| *LBX1* | 0.00021 ± 0.000099 | 0.00027 ± 0.000086 | | 0.003 | 0.00029 ± 0.00012 | 0.00031 ± 0.00013 | 0.46 |
| *MyoD* | 0.00198 ± 0.00070 | 0.00277 ± 0.00123 | | 0.005 | 0.00282 ± 0.00117 | 0.00311 ± 0.00138 | 0.43 |

AIS patients were found to have remarkably lower mRNA expression of *LBX1* and *MYOD* in the concave side than in the convex side. For CS patients, there was no significant difference regarding mRNA expression between the concave side and the convex side. The average mRNA expression of *LBX1* was remarkably lower in AIS patients than in CS patients.

**Supplementary Table 4 Comparison of muscle fiber features between AIS and CS**

|  | **AIS** | |  | **CS** | |
| --- | --- | --- | --- | --- | --- |
|  | **Concave** | **Convex** |  | **Concave** | **Convex** |
| CSA (μm^2^) | 1250.2 ± 204.7 | 1452.4 ± 301.2 |  | 1681.2 ± 391.8 | 1743.2 ± 354.5 |
| Proportion of Type I fiber | 44.4% ± 12.7% | 65.4% ± 16.3% |  | 67.4% ± 21.2% | 79.5% ± 25.2% |

The average cross-sectional area (CSA) of muscle fibers was remarkably smaller in AIS than that in CS (p = 0.02). There was significantly less type I fiber in the concave muscles as compared with the convex muscles in AIS patients (p = 0.004). There was no significant difference regarding the proportion of fiber type between the bilateral sides of paraspinal muscles in CS patients.

**Supplementary Table 5 Results of CCK8 test for MSCs isolated from AIS and CS patients**

| **h** | **AIS** | | | | | |  | **CS** | | | | | |
| --- | --- | --- | --- | --- | --- | --- | --- | --- | --- | --- | --- | --- | --- |
|  | **Concave** | | | **Convex** | | |  | **Concave** | | | **Convex** | | |
| 0 | 0.495 | 0.513 | 0.564 | 0.544 | 0.556 | 0.537 |  | 0.592 | 0.525 | 0.603 | 0.537 | 0.621 | 0.591 |
| 24 | 0.681 | 0.744 | 0.677 | 0.705 | 0.768 | 0.721 |  | 0.768 | 0.782 | 0.724 | 0.781 | 0.724 | 0.754 |
| 48 | 0.941 | 1.025 | 0.984 | 0.987 | 1.145 | 1.084 |  | 1.117 | 1.142 | 1.165 | 1.138 | 1.196 | 1.107 |
| 72 | 1.263 | 1.375 | 1.294 | 1.537 | 1.461 | 1.487 |  | 1.571 | 1.566 | 1.627 | 1.566 | 1.625 | 1.673 |

MSCs cells isolated from the AIS muscles showed obviously lower viability than those of CS patients.

**Supplementary Table 6 Comparison of fusion rate between AIS MSCs and CS MSCs**

|  | **AIS (n=5)** | **CS (n=5)** | P |
| --- | --- | --- | --- |
| 5^th^ Day | 10.93% ± 0.71% | 15.73% ± 1.51% | <0.001 |
| 8^th^ Day | 17.01% ± 2.81% | 24.01% ± 3.05% | <0.001 |

Remarkably lower fusion index of the myotube was observed for AIS group at the 5th day and the 8th day as compared with the CS group.

**Supplementary Table 7 Results of CCK8 test for MSCs transfected with lentivirus *LBX1***

| **h** | ***shLBX1*** | | | ***shCtrl*** | | | ***Control*** | | |
| --- | --- | --- | --- | --- | --- | --- | --- | --- | --- |
| 0 | 0.525 | 0.537 | 0.563 | 0.526 | 0.511 | 0.535 | 0.521 | 0.562 | 0.588 |
| 24 | 0.641 | 0.617 | 0.638 | 0.634 | 0.678 | 0.689 | 0.675 | 0.692 | 0.701 |
| 48 | 0.774 | 0.762 | 0.822 | 0.857 | 0.824 | 0.822 | 0.878 | 0.835 | 0.849 |
| 72 | 0.898 | 0.935 | 0.924 | 1.190 | 1.040 | 1.010 | 1.210 | 1.170 | 1.090 |

Significantly inhibited proliferation rate of MSCs was observed in the *ShLBX1* group as compared with the other two groups.

**Supplementary Table 8 Correlation between the expression of myogenic markers with *LBX1* in paraspinal muscles**

|  | **r** | **P** |
| --- | --- | --- |
| ***MyoD*** | 0.57 | 0.0001 |
| ***MyoG*** | -0.11 | 0.61 |
| ***MYF6*** | -0.18 | 0.42 |

There was significant correlation between the mRNA expression of *LBX1* and *MyoD.* As for the other two myogenesis-associated genes, no correlation with *LBX1* expression was found. The Spearman correlation analysis was used.
